# Complement Depletion Deteriorates Clinical Outcomes of Severe Abdominal Sepsis: A Conspirator of Infection and Coagulopathy in Crime?

**DOI:** 10.1371/journal.pone.0047095

**Published:** 2012-10-16

**Authors:** Jianan Ren, Yunzhao Zhao, Yujie Yuan, Gang Han, Weiqin Li, Qian Huang, Zhihui Tong, Jieshou Li

**Affiliations:** Department of Surgery, Jingling Hospital, Medical School of Nanjing University, Nanjing, P. R. China; D'or Institute of Research and Education, Brazil

## Abstract

**Background:**

The complement depletion commonly occurred during sepsis, but it was often underestimated compared with severe infection or coagulation dysfunction.

**Objective:**

This study was designed to investigate the alteration of complement system in patients with severe abdominal sepsis and evaluate the role of complement depletion in prognosis of such patients. The relationship between complement depletion and infection or coagulopathy was also explored.

**Methods:**

Forty-five patients with severe abdominal sepsis were prospectively conducted among individuals referral to SICU. Currently recommended treatments, such as early goal-directed resuscitation, source control and antibiotics therapy, were performed. Acute physiology and chronic health evaluation II (APACHE II) and sepsis related organ failure assessment (SOFA) scores were employed to evaluate severity. Plasma levels of C3, C4, CRP, PCT, D-dimer and other parameters were detected within eight times of observation. The 28-day mortality, length of stay, and postoperative complications were compared between complement depletion and non-complement depletion groups.

**Results:**

Within the study period, eight (17.8%) patients died, five of them suffering from complement depletion. The overall incidence of complement depletion was 64.4%. At admission, mean complement C3 and C4 levels were 0.70 and 0.13 mg/mL, respectively. Using ROC analysis for mortality prediction, the area under the curve of C3 was 0.926 (95% CI, 0.845–0.998, P<0.001), with optimal cutpoint value of 0.578 mg/mL. Complement C3 depletion was shown to be no correlation to severity scores, however, strongly correlated with elevated D-dimer, PCT concentrations and increased postoperative complications.

**Conclusions:**

Complement C3 depletion was found to be connected to poor prognosis in severe abdominal sepsis. This depletion seems to be associated with coagulopathy and aggravated infection during sepsis, which should be paid close attention in critical care.

**Trial Registration:**

ClinicalTrials.gov NCT01568853

## Introduction

Abdominal sepsis is commonly accompanied and becomes a major problem for patients who suffered from gastrointestinal fistula [1]. Many of those patients would develop septic shock if infection from various enterogenic microorganisms was poorly uncontrolled. A typical characteristic of sepsis is the dysregulation of innate immunity, the complement system involved due to its crucial role against common pathogens [2]. In fact, the complement system does not only affect robust innate immune responses, but also plays roles in adaptive immunity regulation during the process of sepsis [3], [4].

There is abundant evidence for complement activation when sepsis presents. Three major pathways, including classical, lectin, and alternative, are involved to activate different biological functions, producing numerous complement anaphylatoxins (C3a, C4a, C5a, etc.) meanwhile [5]. The effects of these anaphylatoxins are still controversial, considering the protective and harmful effects in sepsis models. While there is great interest in complement by-products in human sepsis, few studies focus on the consumption of complement components and its roles in outcomes of sepsis. Component C3 is an indispensable community mediator for three canonical pathways of complement activation. In a way, changes of C3 levels could indirectly reflect the whole status of complement functions.

In clinical practice, most of patients with severe sepsis could develop coagulopathy very soon [6]. The activation of complement system is tightly connected with hemostasis during sepsis [7]. However, to our knowledge, few human data have investigated the detailed interconnections between complement and coagulation in severe sepsis. Furthermore, the incidence of complement depletion in severe sepsis was seldom reported, and its role in predicting mortality was also poorly understood.

Hence, the purpose of this prospective study was two-fold: first, to explore the incidence of complement depletion and its associated outcomes in a cohort of patients with severe abdominal sepsis; and second, to investigate the relationship between complement system with coagulation dysfunction and inflammation by observing the alterations of complement components and relevant parameters during the process of sepsis. All taken together serve to evaluate the application of C3 related-indexes in predicting the prognosis of such sepsis.

## Methods

### Study Design and Ethics

This was a prospective pilot study of a cohort of patients with severe abdominal sepsis at the Surgical Intensive Care Unit (SICU) of a tertiary level hospital in China. This facility has 37 beds, and provides critical care services for about 1500 patients each year. The period of this study was from November 2011 to March 2012.

The study was approved by the ethics committee of Jinling Hospital and registered by ClinicalTrials.gov (NCT01568853). Written informed consents were obtained from all subjects or their closest relatives. The main contents of informed consents included as follows: 1. The nature of all procedures, including the reason why the patient was suggested to join this study, when and how medicine would be prescribed and timing of sample collections; 2. The relevant benefits, risks and uncertainties related to interventions; 3. The assessment of understanding and acceptance of interventions by the patient. The protocol for this trial is available as supporting information; see Protocol S1.

### Study Setting and Population

The study cohort included patients over 18 years of age who suffered from severe abdominal sepsis. Patients would be enrolled into this study if they conformed to the following criteria: 1) confirmed diagnosis of gastrointestinal fistula through CT-scan or gastroenterography; 2) at least two of four criteria of systemic inflammatory response syndrome (SIRS) complicated with organ dysfunction, hypoperfusion (mean arterial pressure (MAP)<60 mmHg after 20 mL/kg crystalloid), or hypotension (systolic blood pressure (SBP)<90 mmHg); 3) severe intra-abdominal infection, with positive culture of enterogenic microorganisms in blood or identifiable site of infection.

Detailedly, SIRS was defined by two or more of the following conditions: temperature >38 or <36°C; heart rate >90 beats/min; respiratory rate >20 breaths/min or PaCO_2_ <4.26 kPa; white blood cell count >12,000 or <4,000 cells/µL (or >10% immature forms). Sepsis was defined as a systemic response to infection including the criteria of SIRS plus microbiological evidence of a focal infection and/or a positive blood culture. Severe sepsis was defined as sepsis associated with organ dysfunction. Septic shock was defined by the persistent presence of sepsis-induced tissue hypoperfusion refractory to adequate fluid resuscitation. Sepsis-induced tissue hypoperfusion was characterized by SBP<90 mmHg or MAP<70 mmHg or a SBP decrease >40 mmHg or <2 SD blow normal for age in the absence of other causes of hypotension [8].

Exclusion criteria were age <18 or >60 years, pregnancy, leucopenia resulted from radiochemical therapy for malignant tumor, any primary diagnosis other than sepsis, confirmed immunodeficiency or coagulation disorders, requirement for blood transfusion (whole blood, erythrocyte, plasma and cryoprecipitate), blood purification (plasmapheresis, CRRT, CVVH, CHDF, BL, etc.), immunosuppressive agents or immediate surgery. Of note, as previously designed, patients who died within 7 days of admission were also excluded [9], [10].

Patients were stratified into two main groups according to the plasma levels of complement C3 after admission. Specifically, patients, who had persistent low levels of complement C3 (less than 0.75 mg/mL) within the first three days of admission, would be categorized into complement depletion group. The rest, that included normal complement C3 or first low level but back to normal soon, would be assigned into control group. The watershed value for complement C3 was the lower limit of the reference interval in our hospital. All therapeutic interventions for sepsis were kept identical between the two groups.

### The Management of severe abdominal sepsis

All enrolled patients had severe sepsis due to high volumes of fistula output or severe intra-abdominal infection from complicated appendicitis, acute pancreatitis, and other primary illnesses. The management for such populations is complicated and stage-by-stage processed. In brief, the standard management of severe sepsis includes: 1. Fluid resuscitation; 2. Norepinephrine (intravenously, 10 µg/min, 2–3 h) as primary vasopressor; 3. Early goal-directed therapy (MAP >65 mmHg, and SvO_2_ >65%); 4. Source control; 5. Antibiotics therapy; 6. Other supporting treatments as needed. For fistula management, the four pivotal principles, outlined by Chapman and colleagues [11], consist of correction of intravascular volume deficit, drainage of abscess, control of fistula, and protection of the skin. No patient was allowed to use standardized unfractionated heparin or anticoagulant factor concentrates in the entire duration of study, without activated protein C (APC) therapy either.

In addition, nutrition therapy (parenteral/enteral nutrition), persistent surgical lavage and drainage, mechanical ventilation (ASV, CMV, IMV, etc.), somatostatin (Merck Serono, UK, 6 mg/day) infusion and other assistant measures were selected to apply for specific patients. Short-acting insulin analogs were administrated subcutaneously to control hyperglycemia for partial cases.

### Complement levels and coagulation evaluation

Samples of peripheral blood were collected into heparinized syringes (Sodium heparinate, Ratiopharm, China) at baseline, 1 d, 2 d, 3 d, 7 d, 14 d, 21 d, and 28 d of admission. Plasma was obtained from the centrifugation (3000 *g*, 20 min, 4°C), immediately stored at −80°C until tested.

The different complement components test measures were chosen according to their different baseline levels, and various sensitivities to measurement. Briefly, complement C3 was measured with the Cobas Integra platform (Roche Diagostics, Ltd., West Sussex, UK), complement C4 by nephelometry (Dade Behring, Milton Keynes, UK). Coagulation indexes, such as prothrombin time (PT), activated partial thromboplastin time (aPTT) and D-dimer, were determined at each time point of observation. Besides, other routine laboratory parameters indicating the evolution of sepsis [white blood cells (WBC), C-reactive protein (CRP), procalcitonin (PCT), etc.] were detected meanwhile.

### Observing indexes and Severity Scores

Blood routine examination, routine stool test, urine routine examination, blood biochemical analysis, blood gas analysis, drug administration (types of vasopressor, dosage, etc.) and other indexes were monitored meanwhile after admission. Those indexes were used for the simplified acute physiology score II (SAPS II), Acute Physiology and Chronic Health Evaluation II (APACHE II) score and Sequential Organ Failure Assessment (SOFA) score [12], [13].

### Outcomes and risk factors

The primary outcome was the 28-day mortality after admission to SICU. Briefly, mortality was defined as any death occurring during the hospital stay. The predicted death rate was calculated based on the APACHE-II death equation [13]. The secondary outcomes included incidence of postoperative complications, duration of ICU stay, length of hospital stay, and hospital charges. The potential risk factors in the assessment, filtered from a univariate logistic analysis with P<0.05 for model entry, included age, sex, body mass index (BMI), primary disease, number of injured organs (based on International Classification of Diseases [ICD]-10 codes upon discharge), SAPS II, APACHE II, SOFA, complement C3, PCT, D-dimer and open abdomen therapy.

### Data Analysis and Statistics

Data were expressed as mean ± standard deviation if no specific statement. A Kolmogorov-Smirnov test will be used to verify the normal distribution of data. A student's *t*-test was performed for two groups' comparisons of continuous parametric variables, with the Mann-Whitney U-test for continuous variables and Fischer's exact test for categorical variables in non-parametric tests. A multivariate logistic regression using the risk factors and the outcomes (primary/secondary) was performed with stepwise method, results reported as odds ratio (OR) and 95% confidential intervals (CIs). Cross-correlation analysis was used to detect the relationship between complement C3 and other parameters mentioned above, with P<0.05 for regression significance. Statistical analyses were performed by using SPSS Software (version 16.0; SPSS inc., Chicago, IL). Receiver operating characteristic (ROC) analysis was constructed by using STATA software (version 12.0 StataCorp., College Station, TX, USA) to evaluate the accuracy of risk prediction comparing the calculated mortality with the actual occurrence. *P* value below 0.05 was considered statistically significant.

## Results

**Figure 1:**
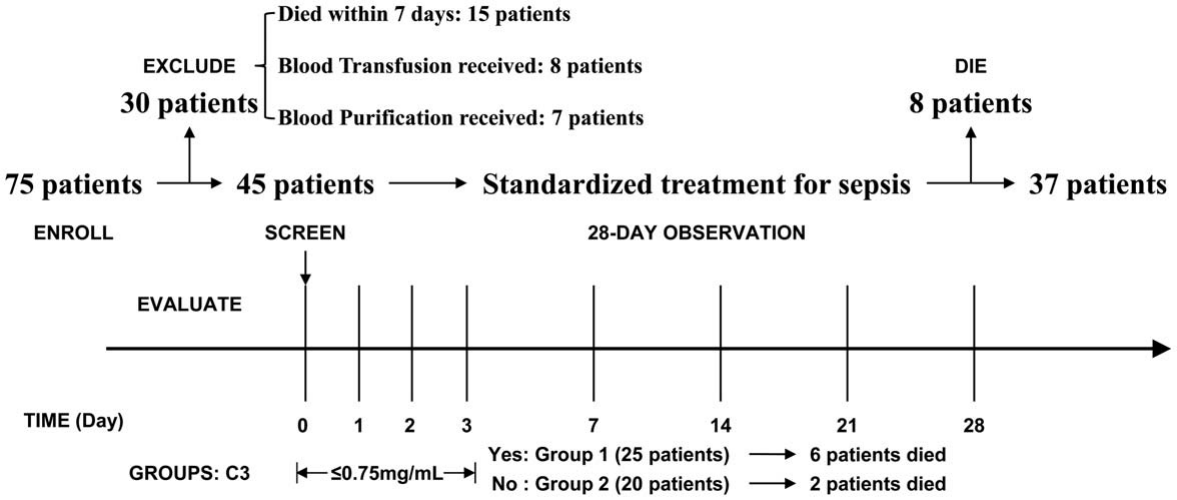
Enrollment and Study design. A total of eight evaluations were performed. Patients were excluded for three medical reasons, including death within seven days after admission, blood transfusion and blood purification within the period of study. All enrolled patients were followed up for 28 days, and 28-day mortality was assessed as the primary outcome.

Seventy-five patients were enrolled during the interval of this study. Generally, all enrolled patients were in accordance with community-acquired sepsis with severe illness. Of this cohort, 30 patients were excluded according to exclusion criteria, with 15 cases died within one week of admission. For those early dead, ten patients died from progressive sepsis, four from multiple organ failure, and one from uncontrolled hemorrhage. A total of 45 patients with severe sepsis were prospectively included in this study (Fig. 1). Based on baseline levels of complement C3 as mentioned above, 25 patients were assigned into complement depletion group (group 1), with the rest into control group (group 2). The general characteristics of the 45 septic patients are shown in Table 1. There was no significant difference in age, gender, BMI, severity scores, WBC, CRP and PCT levels between the two groups, but C3 and C4 baseline levels in group 1 were significantly lower than those in group 2. Besides all patients in group 1, an additional four patients had complement depletion at admission, but back to normal within three days. Hence, the incidence of complement depletion in severe sepsis was about 64.4%. At the end of study, nine (20.0%) patients from group 1 still had complement depletion.

**Table 1:** Subject's descriptive characteristics.

Variable*	Pooled (n = 45)	Group 1 (n = 25)	Group 2 (n = 20)	*P* a
Age (yr)	43.8 (13.2)	44.3 (11.2)	43.2 (10.6)	0.532
Gender male	82%	80%	85%	0.716
BMI	21.2 (2.1)	20.9 (2.3)	21.6 (2.1)	0.327
Mortality [n (%)]	8 (18)	6 (24)	2 (10)	0.269
Primary Disease [n]				
SAP	11	5	6	0.5
Selective Operation	17	10	7	0.767
Trauma	11	7	4	0.729
Necrotizing fasciitis	2	2	0	-
Others b	4	1	3	0.309
APACHE II (24 h)	17.1 (1.7)	17.3 (1.5)	16.8 (1.7)	0.128
SAPS II (24 h)	39.7 (9.1)	40.2 (8.9)	38.2 (10.3)	0.088
SOFA (24 h)	9.8 (1.3)	10.0 (1.1)	9.6 (1.4)	0.205
C3 (mg/mL)	0.70 (0.31)	0.50 (0.18)	0.95 (0.24)	<0.001
C4 (mg/mL)	0.13 (0.06)	0.04 (0.03)	0.25 (0.14)	<0.001
CRP (mg/L)	196.1 (35.7)	217.3 (49.6)	199.5 (31.3)	0.074
PCT (ng/mL)	11.1 (2.9)	11.3 (2.8)	10.8 (3.1)	0.126
WBC (×10^9^/L)	16.1 (2.1)	16.3 (2.0)	15.8 (2.1)	0.206

* Data present as mean (SD) if no specific statement. BMI, body mass index; SAP, severe acute pancreatitis; APACHE II, acute physiology score and chronic health evaluation II; SAPS II, simplified acute physiology score II; SOFA, sequential organ failure assessment score; CRP, c-reactive protein; LOS, length of stay;

a by *Mann-Whitney U test* or *Fischer exact test*;

b include acute appendicitis, acute suppurative cholangitis, and acute gastric perforation.

### Outcomes of sepsis

Within the period of 28-day observation, eight (17.8%) of 45 patients died of progressive disease: five from pneumonia, two from uncontrolled intra-abdominal bleeding, and one from multiple organ failure. Except of two patients who died from pneumonia, other non-survivors suffered from severe complement C3 depletion after admission. Compared with the survivors, the dead subjects shared the similar features in respect to age, sex, and etiology, but had markedly lower baseline levels of C3 (0.36±0.18 vs. 0.78±0.27, *P*<0.001), and higher APACHE II (18.1±1.1 vs. 16.2±1.7, *P* = 0.004) and SOFA scores (11.0±0.9 vs. 9.3±1.2, *P*<0.001). The predicted death rate for the study was 25.0% and the observed death rate was 17.8%.

Definitive operation, used to eliminate the source of infection, was performed for all patients. The mean interval from admission to a definitive operation was 16.3±6.3 (range, 6–24) days. The difference of the interval between the two groups was significant (21.1±10.7 vs. 10.3±3.6 days, *P*<0.001).

The postoperative complications occurred in nine (20%) patients during the observation period (Table 2). The incidence of complications was increased in complement depletion group compared with control group, without significant difference (*P* = 0.260). Patients were more susceptible to pneumonia than other types of complications. Although the difference of mortality in hospital or SICU was not significant, the length of stay in both hospital and SICU was markedly extended in complement depletion patients compared with control patients (Table 2). Similarly, the mean hospital charges in complement depletion group were greatly higher than those in control group ($35, 005±$8, 667 vs. $25, 486±$11, 810, *P* = 0.024).

**Table 2:** Outcomes after 28-day observation in two groups.

Variable*	Pooled (n = 45)	Group 1 (n = 25)	Group 2 (n = 20)	*P* a
28-day mortality [n (%)]	8 (18)	6 (24)	2 (10)	0.269
ICU mortality [n (%)]	7 (16)	5 (20)	2 (10)	0.437
Hospital LOS (days)	25.3 (7.1)	28.2 (7.2)	21.3 (6.3)	0.008
ICU LOS (days)	8.2 (2.4)	9.7 (2.6)	6.3 (1.1)	<0.001
Open abdomen [n (%)]	6 (13)	5 (20)	1 (5)	0.205
Postop complications [n]	9	7	2	0.260
Surgical site infection	4	3	1	0.617
Pneumonia	7	6	1	0.112
Urinary tract infection	1	1	0	-
Hemorrhage	4	3	1	0.617
Incisional hernia	1	1	0	-

* Data present as mean (SD) if no specific statement. LOS, length of stay; Postop, postoperative;

a by *Mann-Whitney U test or Fischer exact test*.

### Relationship between complement C3 depletion and severity scores

To investigate the role of complement depletion in prediction of mortality for such patients, ROC curve analysis with conventional APACHE II, SOFA, SAPS II scores and C3 baseline levels were constructed from the predicted and observed outcome of sepsis (Table 3). The area under the curve (AUC) of complement C3 was much larger than that through any severity scores. Besides, the optimal cutpoint of C3 for predicting 28-day mortality was 0.578 mg/mL, with sensitivity 78.4% and specificity 99.8%.

**Table 3:** Receiver operating characteristic (ROC) curve analysis in prediction of mortality.

Variable	AUC (CI)	Cut-off value*	Sn/Sp (%)
APACHE II score	0.824 (0.700–0.949)	>16	65/100
SOFA score	0.853 (0.732–0.974)	>10	86/62
SAPS II score	0.823 (0.692–0.953)	>41	81/75
C3 levels (mg/mL)	0.926 (0.845–0.998)	<0.578	78/100

AUC area under curve by Hanley method to calculate standard errors, CI 95% confidential intervals, Sn/Sp sensitivity/specificity, APACHE acute physiology and chronic health evaluation, SOFA sequential organ failure assessment, SAPS II simplified acute physiology score II.

* For the severity scores, higher values are predictors of mortality; but for the C3 levels, lower values are predictors of mortality.

**Figure 2:**
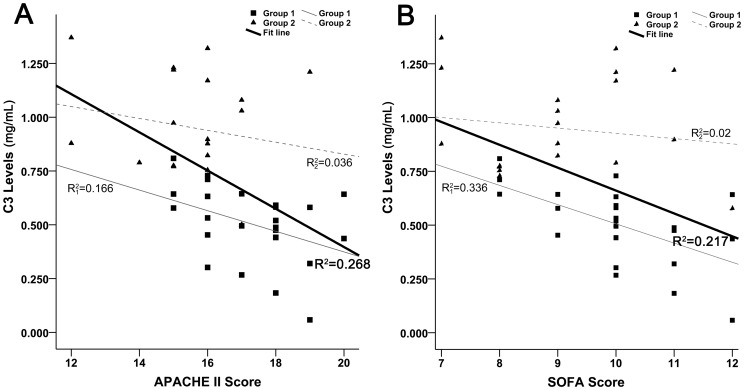
The correlation analysis between baseline levels of C3 and severity scores. (A) The relationship between C3 and APACHE II score. Data in both groups were pooled for analysis (n = 45). The linear regression indicates that the baseline levels of C3 have little correlation with APACHE II score (R^2^ = 0.268). (B) The relationship between C3 and SOFA score. Data in all patients were included for analysis (n = 45). The regression result shows that C3 have no correlation with SOFA score (R^2^ = 0.217). In addition, linear regression for each group was performed, with similar results to the pool.

The baseline levels of C3 are not associated with either APACHE II or SOFA scores for such populations (Fig. 2). In addition, a similar result is obtained for complement C4, with R^2^ = 0.155 for APACHE II score and R^2^ = 0.014 for SOFA score. In a way, the complement depletion in early stage of sepsis may be considered as an independent risk factor in predicting clinical outcome.

### The changes of complement components, inflammation and coagulation indexes

**Figure 3:**
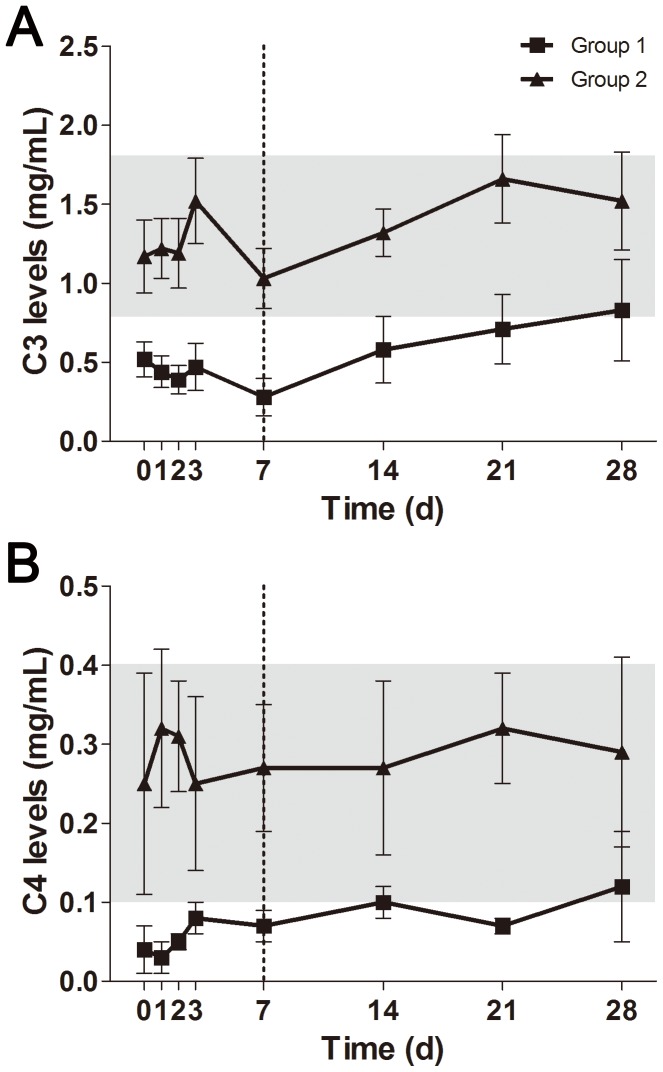
The observed changes of complement components in management of sepsis. These dynamic changes were directly compared between group 1 and group 2. Plasma levels of C3 (A) and C4 (B) within 28-day observation were quite different between both groups (*P*<0.001). The gray area indicates the normal reference range. Data present as mean ± SD.

**Figure 4:**
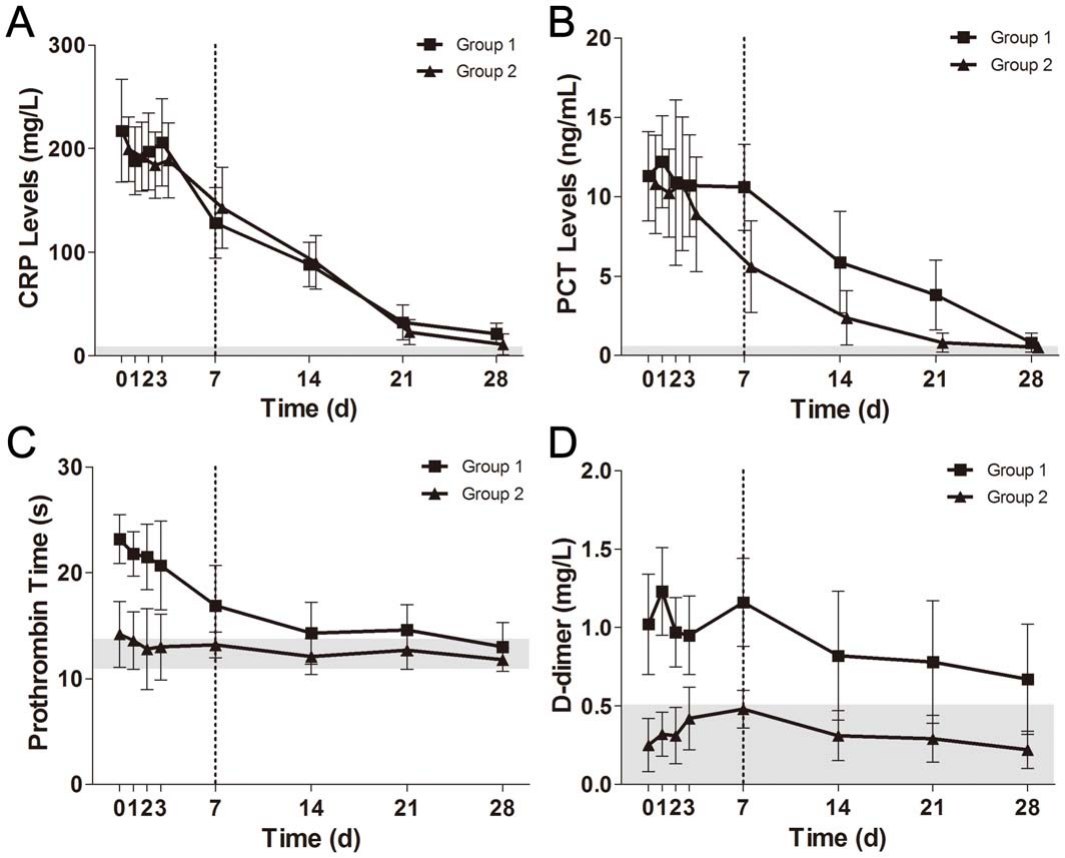
The observed changes of infection and coagulation indexes in management of sepsis. The values of each index were compared between group 1 and group 2. All data present as mean ± SD. CRP (A) and PCT (B) were utilized to evaluate the inflammatory response to sepsis. PT (C) and D-dimer (D) were employed to evaluate the coagulation function during sepsis. The gray area indicates the normal reference range.

As mentioned in Methods, we divided our patients into two equal groups according to the baseline levels of C3 within the first three days of admission. The complement depletion, which included C3 and C4 depletion, was almost impossible to recover back to normal once it occurred in the early phase of sepsis (Fig. 3). There was a significant difference in overall levels of complement C3 and C4 between the two groups (*P*<0.05). The current management strategies did little contribution to concentration improvement of complement components. Briefly, the levels of CRP and PCT were decreased in both groups, but no significant difference observed (Fig. 4A). However, it seemed that the reduction of PCT concentration in complement depletion patients was delayed as compared with control patients. As for the coagulation indexes, prothrombin time and D-dimer concentration were increased in group 1 compared to group 2, with significant difference before 7 days of admission (Fig. 4B).

### Linear regression and Multivariate logistic regression analysis

Linear regression analysis by stepwise method confirmed a significant relationship between complement C3 with inflammation and coagulation (Table 4). Specifically, for every unit decrease in C3 plasma level, there is a 1.56 mg/L increase in D-dimer concentration (95% CI, 1.13–1.98; *P*<0.001) and a 8.5 ng/mL increase in PCT concentration (95% CI, 6.9–10.1; *P*<0.001), respectively. In addition, the complement depletion in early stage of sepsis was further associated with prolonged length of stay in both ICU (*P*<0.0001) and hospital (*P*<0.0001). Using multivariate logistic regression, we sought to identify predictors of poor outcomes of sepsis. Age, severity scores, C3 levels, and D-dimer concentration were strongly associated with the mortality. In addition, complement C3 depletion was correlated with increased odds of pneumonia and hemorrhage (Table 5). Taken together, these results indicate that the early exhaustion of complement components is strongly associated with worse outcomes in sepsis patients. Nevertheless, the slow recovery of physiologic levels of complement C3/C4 under current management strategies might explain this increased incidence of pneumonia or hemorrhage.

**Table 4:** Univariate linear regression between C3 and coagulation and infection.

Predictor	Dependent	R	B	CI	*P*
C3	PT (s)	0.289	−2.153	−(0.128–4.179)	0.038
C3	D-dimer (mg/L)	0.722	−1.556	−(1.133–1.979)	<0.001
C3	aPTT (s)	0.319	3.869	0.601–7.137	0.021
C3	Fib (mg/dL)	0.277	101.6	1.672–201.6	0.046
C3	INR (ratio)	0.043	−0.018	−0.134–0.099	0.760
C3	CRP (mg/L)	0.155	−24.85	−69.86–20.17	0.273
C3	PCT (ng/mL)	0.831	−8.543	−(6.918–10.16)	<0.001

PT, prothrombin time; aPPT, activated partial thromboplastin time; Fib, fibrinogen; INR, international normalized ratio; CRP, c-reactive protein; PCT, procalcitonin; R, correlation coefficient; B, slope of the regression (also called regression coefficient). CI stands for 95% confidential intervals.

**Table 5:** Complement C3 depletion and sepsis outcomes.

Predictor	Dependent	OR	SE	CI	*P*
C3	Mortality	0.934	0.761	0.189–4.611	0.034
C3	ICU mortality	0.947	0.675	0.168–4.231	0.013
C3	Pneumonia	0.537	0.445	0.106–2.723	0.006
C3	Hemorrhage	0.255	0.223	0.046–1.414	0.029

Logistic regression performed by using the baseline levels of C3 at admission of SICU; OR, odds ratio; SE, standardized error; CI stands for 95% confidential intervals.

## Discussion

In this prospective study, we stratified enrolled sepsis patients into two main groups based on baseline levels of complement C3, rather than conventional APACHE II score. The first group consisted of patients with complement C3 levels less than 0.75 mg/mL, with mean levels of 0.50 mg/mL at admission to SICU. Within 28-day observation, six patients died in this group, as compared with two patients in control group. The severity scores (APACHE II, SAPS II, and SOFA) had no significant difference between the two groups. The early complement C3 depletion, as an independent risk, had better effect than classic severity scores on prediction of mortality for septic patients. Besides, the strong association between complement C3 depletion and coagulopathy was observed, with a distinct relationship to D-dimer concentration during sepsis. The exhaustion of complement components also contributed to the increased incidence of postoperative complications subsequently.

Of note, patients who underwent blood transfusion or blood purification were excluded from this study. Generally, blood transfusion, in particular fresh frozen plasma (FFP), would provide additional complement components for the recipient [14]. Although this treatment was helpful for patients with declined or depleted complement concentrations, it must conceal the real incidence of complement depletion in septic or septic shock patients [15]. Besides, there was substantial evidence that various types of blood purification could consume complement components (C3, C4, C5, etc.) during the removal of certain inflammatory cytokines [16]–[18].

In clinical practice, the complement depletion is often underestimated or even ignored, as compared with severe sepsis, hemorrhage, and other critical illness. In this study, the incidence of complement C3 depletion was 64.4%, with 20.0% (9 patients) at the end of observation. This high incidence was simply underestimated, but related to coagulation dysfunction [19].

Coagulopathy, in particular hypocoagulability, is a common complication after severe sepsis [6]. For decades, it was generally recognized that any hypocoagulable status after sepsis was due to iatrogenic pathogenesis. In brief, hypothermia, volume dilution, and metabolic acidosis were currently accepted consensus to explain the emergence of coagulopathy. Moreover, the recent studies have indicated that the complement depletion, such as C3, C1q and B factor, is strongly associated with the incidence of coagulopathy during sepsis [20], [21].

In fact, complement components contribute significantly to thrombosis by directly strengthening blood clotting properties and by augmenting the inflammatory response, which subsequently enhance coagulation reaction [22]. The most highly studied clinical condition wherein coagulation dysfunction and complement-related inflammation coexist is sepsis. As sepsis progresses, complement activation by considerable release of cytokines (TNF-α, IL-6, IL-10, etc.) produced enormous components, such as C3a, C4a, and C5a, and further consumed the pool of complement C3 and C4 [23]. As for the observed correlation between D-dimer and C3, it can be explained through the bridge of CRP. CRP directly influences several stages of sepsis via complement activation, lipid accumulation and thrombosis [24], [25]. Strong correlations were confirmed in D-dimer concentrations with circulating CRP levels [26]. Besides, D-dimer alone was reported as an early prognostic marker of the evolution and complications of certain abdominal sepsis [27]. However, the concrete mechanism remains obscured, and needs some further studies in-depth to unravel their relationship.

Although APACHE II system was found to be meaningful in outcome prediction of sepsis, the diverse values of AUC found by various studies indicated it was not specific or precise enough for mortality prediction of critically ill patients [28]–[32]. Recent studies have demonstrated that extensive complement activation and subsequent complement depletion played essential role in weakening coagulation function during sepsis [5], [20]. This compromising effect would cause DIC and subsequent MODS, adversely affects the outcome of sepsis. Moreover, the exhaustion of complement C3 in late stage of sepsis was associated with significantly decreased defense to pathogenic invasion due to injured T-cell immunity [33]. Thus, complement C3 depletion could potentially reflect the severity and prognosis of septic patients. In current study, the AUC of ROC curve for mortality prediction was 0.926, better than general severity scores. More importantly, our finding indicated that C3 level was not correlated with such classical severity scores, which was quite different from other prognostic biomarkers such as CRP and IL-6.

Moreover, there is also evidence indicating that the elevated PCT concentrations are associated with complement activation and depletion, in particular C3a [34]. High levels of PCT were first described in children with severe bacterial infections, and were suggested to be a specific marker for bacterial infection [35]. Importantly, the combination of increased C3a and PCT concentrations can be excellently used to discriminate patients from the sepsis to the SIRS [34].

Pulmonary neuroendocrine cells in the bronchial epithelium has been reported to be a major source of calcitonin immunoreactivity [36], [37]. In our study, the observed complement depletion may increase the risk of pulmonary infection by weakening the innate immunity. That would be reasonable to explain the destroyed structure of bronchial epithelium and its associated release of PCT into circulation. Based on our experimental results, extensive lung damages due to severe sepsis were significantly distinct, associated with the exhaustion of complement C3 [38]. However, we also found that CRP was not sensitive as PCT in accordance with complement C3 consumption, which may indicate the important role of complement in late stage of severe sepsis.

There are several limitations and questions raised by current observational study. First, the relatively small sample size would depress statistical power and bring in some uncertainty to the conclusion. The large 95%CI of the ORs in Table 5 actually reflect the low number of events per covariable. Besides, another important limitation of this study is lack of generalizability for sepsis. Most of enrolled patients developed sepsis from severe intra-abdominal infection rather than other infectious sources (pulmonary infection, urinary tract infection, meningitis, etc.). Therefore, it would be insufficient to generalize the findings to sepsis at large. Finally, the complement C3a was failed to investigate since numerous studies have confirmed its extensive expression in sepsis and associated effects as a predictor for outcome of sepsis. Other complement components should be tracked during sepsis to better understand the whole function of complement system. Of course, another study combining with anticoagulation therapies should be considered in future study.

In conclusion, the results of this study demonstrate that complement C3 depletion could indicate poor clinical outcomes in severe abdominal sepsis. Such depletion seems to be an independent risk factor in predicting mortality. With the small sample size, the reliable prediction is uncertain, and remains to be confirmed in further rigorous studies. Moreover, the findings suggest a correlation between C3 depletion with elevated D-dimer and PCT concentrations. The complement depletion should be paid close attention and further confirmed in critical care patients.

## Supporting Information

Protocol S1 **Trial Protocol.**
(PDF)
