# Correction: Complement Depletion Deteriorates Clinical Outcomes of Severe Abdominal Sepsis: A Conspirator of Infection and Coagulopathy in Crime?

**DOI:** 10.1371/annotation/45250927-10e7-4545-8fdf-066e688cc125

**Published:** 2013-03-22

**Authors:** Jianan Ren, Yunzhao Zhao, Yujie Yuan, Gang Han, Weiqin Li, Qian Huang, Zhihui Tong, Jieshou Li

There is an error in the affiliation shared by all authors. The affiliation should state:

Department of Surgery, Jinling Hospital, Medical School of Nanjing University, Nanjing, P.R. China 

