# Supplementary material for: Complement Depletion Deteriorates Clinical Outcomes of Severe Abdominal Sepsis: A Conspirator of Infection and Coagulopathy in Crime?
Source: PLoS One. 2012 Oct 16;7(10):e47095. doi: 10.1371/journal.pone.0047095 (PMC3473032; doi:10.1371/journal.pone.0047095)
Supplement: Protocol S1 — Trial Protocol. (PDF) [file pone.0047095.s001.pdf]

Protocol Registration Receipt
04/05/2012

A Prospective Study of The Complement Depletion in Patients With Severe Abdominal Sepsis

This study has been completed.

Sponsor:	Jinling Hospital, China
Collaborators:	
Information provided by (Responsible Party):	Jianan Ren, Jinling Hospital, China
ClinicalTrials.gov Identifier:	NCT01568853

Purpose

The role of complement system in bridging innate and adaptive immunity has been confirmed in various invasive pathogens. The aim of this study is to investigate the alteration of complement C3 in patients with severe abdominal sepsis and evaluate the role of complement C3 depletion in prognosis of such patients. The relationship between complement C3 depletion and adaptive immunity is studied meanwhile.

Condition	Intervention	Phase
Severe Sepsis<br>Pancreatitis<br>Abdominal Abscess<br>Appendicitis<br>Digestive System Fistula	Drug: Norepinephrine<br>Procedure/Surgery: Open abdomen<br>enteral nutrition<br>parenteral nutrition	N/A

Study Type: Interventional
Study Design: Screening, Single Group Assignment, Open Label, N/A, N/A
Official Title: The Complement C3 Depletion in Patients With Severe Abdominal Sepsis: Risk Prediction and the Association With Down-regulated Adaptive Immunity

Further study details as provided by Jianan Ren, Jinling Hospital, China:

Primary Outcome Measure:

- All cause mortality [Time Frame: within the first 28 days after admission to our hosptial] [Designated as safety issue: No]
  Patients died within the first three days of admission would be excluded from this study.

Secondary Outcome Measures:

- Postoperative complications [Time Frame: within the first 28 days after admission to our hosptial] [Designated as safety issue: No]
  wound complications; pulmonary infection; incisional hernia, and bleeding.

Enrollment: 75
Study Start Date: November 2011
Study Completion Date: March 2012
Primary Completion Date: March 2012

Number of arms: 1
Intervention Details:

Drug: Norepinephrine
Intravenously, 10 ug/min, 24 hours

Procedure/Surgery: Open abdomen
IAH >=20 mmHg, and ACS emerged, such as low urine and decreased FiO2 quickly.

enteral nutrition
500-1500 kcal/day; Nasogastric tube feeding;

Other Names:
Peptison (SP; Nutricia, Shanghai, China)

parenteral nutrition
3000 mL parenteral nutrition fluid, intravenously.

Other Names:
Made by our hospital.

Severe abdominal sepsis remains a significant cause of death in patients undergoing intra-abdominal infection, in despite of recent declines in overall mortality. There is a abundant evidence to suggest complement activation during sepsis. While there is great interest in complement by-products in human sepsis, few studies focus on the persistent consumption of complement components and its role in prognosis of sepsis. Complement C3 is indispensable community pathway for complement activation. In a way, the alteration of C3 levels can affect the whole status of complement biological functions.

In clinical practice, the severe abdominal sepsis would develop compromised immune function if the intra-abdominal infection is not well controlled. The down-regulated T- and B-cell immune responses to sepsis are correlated to the decreased immune defense. To our knowledge, there are few human data that have investigated the relationship between complement depletion and adaptive immunity in severe abdominal sepsis. The investigators hypothesize that the complement C3 depletion during sepsis has a stronger association with the

down-regulated adaptive immunity and can be regarded as a essential risk factor to predict the prognosis of such critical illness.

The purpose of this prospective study is two-fold. First, the investigators observe, in a cohort of patients with severe abdominal sepsis, the levels of complement components and percentages of T cell subsets after admission to evaluate the relationship between complement system and adaptive immunity. Second, the investigators also evaluate the application of the C3 related-indexes (C3, C3a, Factor H, DAF, etc.) to patients undergoing severe abdominal sepsis and to develop an alternative model to predict its prognosis.

Eligibility

Ages Eligible for Study: 18 Years to 60 Years
Genders Eligible for Study: Both

Inclusion Criteria:

- Clinical diagnosis of severe abdominal sepsis

Exclusion Criteria:

- Age < 18 or > 60 years
- Pregnancy
- Leucopenia from radiochemical therapy due to malignant tumor
- Any primary diagnosis other than sepsis
- Confirmed immunodeficiency
- Requirement for blood transfusion, plasmapheresis, or immediate surgery

Contacts and Locations

Locations

China, Jiangsu

Department of Surgery, Jinling Hospital
Nanjing, Jiangsu, China, 210002

Investigators

Principal Investigator:	Jianan Ren, M.D.	Department of Surgery, Jinling Hospital, China

More Information

Responsible Party:	Jianan Ren, Clinical professor, Principal investigator, Jinling Hospital, China
Study ID Numbers:	BK2010-017-1
Health Authority:	United States: Institutional Review Board; China: State Food and Drug Administration
